# Which Approach to Choose to Counteract Musculoskeletal Aging? A Comprehensive Review on the Multiple Effects of Exercise

**DOI:** 10.3390/ijms26157573

**Published:** 2025-08-05

**Authors:** Angela Falvino, Roberto Bonanni, Umberto Tarantino, Virginia Tancredi, Ida Cariati

**Affiliations:** 1 Department of Biomedicine and Prevention, “Tor Vergata” University of Rome, 00133 Rome, Italy; angelafalvino95@gmail.com; 2 Department of Biotechnological and Applied Clinical Sciences, University of L’Aquila, 67100 L’Aquila, Italy; 3 Catholic University “Our Lady of Good Counsel”, 1000 Tirana, Albania; umberto.tarantino@uniroma2.it; 4 Department of Systems Medicine, “Tor Vergata” University of Rome, 00133 Rome, Italy; tancredi@uniroma2.it (V.T.); ida.cariati@uniroma2.it (I.C.); 5 Centre of Space Bio-Medicine, “Tor Vergata” University of Rome, 00133 Rome, Italy

**Keywords:** aging, musculoskeletal system, physiology, senescence, senotherapeutic, physical exercise, prevention

## Abstract

Aging is a complex physiological process that profoundly affects the functionality of the musculoskeletal system, contributing to an increase in the incidence of diseases such as osteoporosis, osteoarthritis, and sarcopenia. Cellular senescence plays a crucial role in these degenerative processes, promoting chronic inflammation and tissue dysfunction through the senescence-associated secretory phenotype (SASP). Recently, senotherapeutics have shown promising results in improving musculoskeletal health. Natural compounds such as resveratrol, rapamycin, quercetin, curcumin, vitamin E, genistein, fisetin, and epicatechin act on key signaling pathways, offering protective effects against musculoskeletal decline. On the other hand, molecules such as dasatinib, navitoclax, UBX0101, panobinostat, and metformin have been shown to be effective in eliminating or modulating senescent cells. However, understanding the mechanisms of action, long-term safety, and bioavailability remain areas for further investigation. In this context, physical exercise emerges as an effective non-pharmacological countermeasure, capable of directly modulating cellular senescence and promoting tissue regeneration, representing an integrated strategy to combat age-related diseases. Therefore, we have provided an overview of the main anti-aging compounds and examined the potential of physical exercise as a strategy in the management of age-related musculoskeletal disorders. Further studies should focus on identifying synergistic combinations of pharmacological and non-pharmacological interventions to optimize the effectiveness of anti-aging strategies and promoting healthier musculoskeletal aging.

## 1. Introduction

The aging population represents one of the main global health challenges, with a significant impact on the incidence of musculoskeletal diseases, including osteoporosis, osteoarthritis, and sarcopenia [1]. These conditions are characterized by a progressive decline in the function and integrity of skeletal and muscle tissues, negatively affecting quality of life and increasing the risk of disability and mortality [2,3]. Cellular senescence appears to contribute primarily to the musculoskeletal deterioration that occurs during aging, causing an irreversible arrest of the cell cycle and the release of inflammatory mediators known as the senescence-associated secretory phenotype (SASP). In fact, senescent cells accumulate in tissues and contribute to extracellular matrix degeneration, chronic inflammation, and tissue dysfunction [4]. Fortunately, the selective elimination of senescent cells through senolytics or the modulation of their secretory phenotype through senomorphics would seem to improve musculoskeletal health and counteract the progression of chronic diseases, opening new avenues for targeted and more effective therapeutic interventions [5].

In this context, the combination of dasatinib and quercetin, two of the most studied senolytics, has shown beneficial effects in reducing senescence in animal models and in clinical studies in humans through the regulation of anti-apoptotic processes in senescent cells [6]. Similarly, navitoclax and UBX0101 have been used, respectively, for the treatment of age-related muscle loss and for the elimination of senescent cells from the joints of patients with osteoarthritis, showing promising potential in counteracting the degenerative processes associated with aging [7,8]. Finally, senolytic activity has been suggested for rapamycin, a mechanistic target of rapamycin (mTOR) inhibitor capable of improving muscle function, and for fisetin, which appears to limit bone loss [9].

On the other hand, senomorphics of biological origin such as resveratrol, curcumin, vitamin E, and genistein have shown positive effects in counteracting age-related musculoskeletal decline by regulating key signaling pathways such as nuclear factor kappa-light-chain-enhancer of activated B cells (NF-κB), mTOR, sirtuin 1 (SIRT1), and estrogen-related receptor alpha (ERRα) [10,11]. Particularly, resveratrol is known to activate the SIRT1/forkhead box O1 (FoxO1) signaling pathway, promoting osteoblastic differentiation and reducing bone resorption [12]. Protective effects against sarcopenia have been associated with curcumin and vitamin E as they are able to reduce oxidative damage and promote muscle regeneration [13]; while genistein appears to preserve bone density, with a potential role in counteracting postmenopausal osteoporosis [14]. Interestingly, panobinostat and metformin are among the most studied synthetic senomorphics for their ability to reduce chronic inflammation associated with age-related diseases [15,16].

However, despite the growing interest in senotherapeutics, further research is needed to fill some existing gaps, including a better understanding of mechanisms of action, long-term safety, and bioavailability. In addition, the complex pathogenesis of musculoskeletal disorders requires an integrated approach combining drug therapies and rehabilitation interventions [17,18]. In this context, physical exercise is known to be a promising strategy in regulating cellular senescence, as it improves musculoskeletal health and reduces the risk of chronic diseases associated with aging [19,20,21]. Recent studies have shown that physical exercise can directly influence several molecular pathways targeted by anti-senescence compounds, acting as a modulator of aging. Particularly, weight-bearing exercises, such as running or weightlifting, are known to stimulate bone formation by promoting the differentiation of mesenchymal stem cells (MSCs) towards the osteoblastic lineage [22]. At the same time, myokines released by skeletal muscle during exercise seem to play a crucial role in slowing down osteocyte senescence and preserving mitochondrial function [23]. Interestingly, certain molecules, such as 5-aminoimidazole-4-carboxamide ribonucleotide (AICAR), have been shown to be effective in counteracting sarcopenia and delaying senescent cellular processes by activating molecular targets involved in the exercise adaption [24]. Furthermore, Englund et al. demonstrated that a structured 12-week exercise program significantly reduces the expression of markers such as p16, p21, tumor necrosis factor-alfa (TNF-α), and other pro-inflammatory mediators known to be involved in cellular senescence [25], confirming physical exercise as an effective intervention to slow down aging-related processes [26].

Overall, considering the growing impact of age-related musculoskeletal disorders and the key role of cellular senescence in their progression, it is necessary to identify targeted therapeutic approaches capable of counteracting the accumulation of senescent cells and their deleterious effects. Importantly, the complexity and multitude of molecular pathways involved in musculoskeletal aging requires the development of therapies tailored to the needs of individual patients, highlighting the need to deepen our knowledge of the effects and targets of senotherapeutics and physical exercise. Therefore, the aim of our review was to (i) provide a comprehensive overview of the effects of natural and synthetic anti-senescence compounds used in the treatment of age-related musculoskeletal diseases; (ii) analyze the role of physical exercise in modulating cellular senescence, evaluating its potential as a complementary strategy to drug therapy to enhance its therapeutic effects; (iii) summarize the main clinical evidence about the efficacy and the safety of senotherapeutics in the context of age-related musculoskeletal disorders.

## 2. Literature Search Strategy

For this comprehensive review, 160 articles were selected from the MEDLINE bibliographic database, published between 1945 (the year the database was created) and 2025. Studies on aging in relation to musculoskeletal disorders and potential pharmacological and non-pharmacological strategies to counteract their progression were included. Particularly, this non-systematic research focused on natural and synthetic compounds capable of modulating the cellular senescence process in conditions such as osteoporosis, osteoarthritis, and sarcopenia. In addition, the effectiveness of physical exercise in regulating the same molecular pathways involved in the action of senotherapeutics was analyzed. Finally, evidence was gathered on the clinical relevance of the compounds analyzed and physical exercise in the context of age-related musculoskeletal disorders. The search strategy was based on the use of various combinations of the following keywords: “musculoskeletal aging”, “senescence”, “senotherapeutics”, “physical exercise”, “osteoporosis”, “osteoarthritis”, “sarcopenia”, “bone”, “muscle”, “resveratrol”, “rapamycin”, “quercetin”, “curcumin”, “vitamin E”, “genistein”, “fisetin”, “epicatechin”, “dasatinib”, “navitoclax”, “UBX0101”, “panobinostat”, “metformin”, and “molecular target”. The process was conducted on a global scale, without geographical or ethnic restrictions. Language filters were applied to eliminate articles not written in English. Finally, clinical evidence was included by applying the clinical trial selection filter.

## 3. Natural Compounds

**Figure 1:**
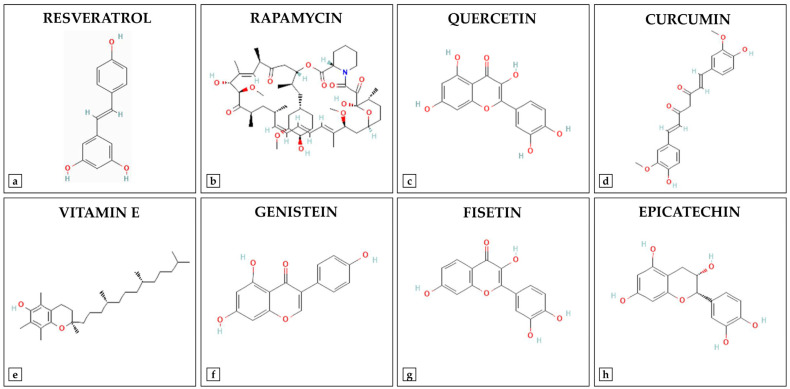
Chemical structure of the main natural compounds with senotherapeutic properties. (**a**) Resveratrol: polyphenolic phytoalexin. (**b**) Rapamycin: macrocyclic lactone. (**c**) Quercetin: flavonoid. (**d**) Curcumin: polyphenol. (**e**) Vitamin E: fat-soluble micronutrient composed of tocopherols and tocotrienols. (**f**) Genistein: isoflavone. (**g**) Fisetin: flavonol. (**h**) Epicatechin: flavonol. Images acquisition from PubChem.

Several natural compounds found in foods or medicinal plants have shown promising senotherapeutic properties thanks to their safety profile and their ability to modulate key processes such as cell survival, oxidative stress and inflammation (Figure 1) [27]. Therefore, their application could offer preventive or complementary strategies to conventional treatments, representing a potential therapeutic resource for the treatment of age-related musculoskeletal disorders (Table 1) [28].

Among these, resveratrol, an edible polyphenolic phytoalexin found in certain plants, has attracted considerable interest for its therapeutic potential in the treatment of senile osteoporosis, thanks to its reductive hydroxylated phenolic group, which gives it antioxidant and anti-aging properties [29]. Its action is mainly manifested through the activation of the SIRT1, promoting the deacetylation and nuclear translocation of FoxO1, resulting in an increase in the antioxidant response [30]. Importantly, because SIRT1 represent a critical center of regulation of cell metabolism, numerous signaling networks and molecular targets are regulated by its activation [31]. In this regard, AMP-activated protein kinase (AMPK) signaling pathway is known to modulate the response to oxidative stress, stimulating extracellular calcification [32]. In addition, resveratrol is known to positively regulate the expression of runt-related transcription factor 2 (RUNX2), a key gene for osteoblast differentiation, through the activation of the SIRT1/forkhead box O3 (FoxO3) axis, promoting osteogenesis and inhibiting adipogenesis [33,34,35]. In this regard, Zhou and colleagues demonstrated that treating senescent MSCs isolated from 12-month-old mice with resveratrol for 48 h reduced the production of reactive oxygen species (ROS) and improved cell viability, as well as promoting extracellular matrix calcification through increased expression of osteogenic genes, such as alkaline phosphatase (ALP), osteocalcin (OCN), osteopontin (OPN), type I collagen (Col-I), and RUNX2. Importantly, pharmacological inhibition of the AMPK signaling pathway significantly attenuated these effects, confirming the central role of resveratrol in mediating the osteogenic response [36]. Similarly, Jiang et al. treated ovariectomized mice with a daily dose of resveratrol for 8 weeks, observing an improvement in bone microarchitecture and an increase in osteogenic markers, such as ALP, RUNX2 and osterix (Osx). Interestingly, treatment of murine osteoblasts subjected to H_2_O_2_-induced oxidative stress with resveratrol promoted cell proliferation and ALP activity, in association with increased superoxide dismutase (SOD) expression and reduced ROS levels, emphasizing the role of resveratrol as an osteoprotective agent [12]. Liao and colleagues studied the efficacy of administering 150 mg/kg/oral resveratrol in counteracting sarcopenia in 25-month-old Sprague-Dawley rats compared to elderly rats of the same age [37]. Analysis of the gastrocnemius muscle showed a significant increase in AMPK, SIRT1, and B-cell lymphoma 2 (Bcl-2) in the treated group, along with a significant reduction in Bcl-2-associated X protein (Bax) and p53 compared to the elderly control rats, highlighting the effectiveness of resveratrol in preventing sarcopenia through the modulation of apoptosis [37].

Rapamycin, a macrocyclic lactone produced by the bacterium Streptomyces hygroscopicus and used primarily as an immunosuppressant, has shown distinct effects on musculoskeletal health [38,39]. Inhibition of mTOR complex 1 (mTORC1) by rapamycin has been shown to be effective in preventing age-related sarcopenia, as chronic activation of this signaling pathway is known to contribute to damage and progressive loss of muscle mass during aging [40,41]. Tang and colleagues reported that hyperactivation of mTORC1 in skeletal muscle fibers increased the content of morphologically abnormal mitochondria and induced progressive oxidative stress, likely by upregulating the expression of FoxO family members and growth differentiation factor 15 (GDF15) through signal transducer and activator of transcription 3 (STAT3) phosphorylation. Importantly, inhibition of mTORC1 with rapamycin reduced GDF15 expression and STAT3 phosphorylation in the skeletal muscle of 30-month-old aged mice, attenuating oxidative stress and muscle fiber loss [42]. On the other hand, Martin et al. administered rapamycin intraperitoneally every other day for 12 weeks to adult wild-type mice and observed a negative impact on bone health. Particularly, the animals showed lower femoral bone mineral content, bone mineral density (BMD) and bone volume than vehicle-treated mice, in association with an increase in serum C-terminal telopeptide of type 1 collagen (CTX-1), a known marker of bone resorption [43]. Notably, the study by Dhanabalan and colleagues suggested rapamycin as a promising strategy for improving osteoarthritis, as the administration of rapamycin-loaded poly(lactic-co-glycolic acid) microparticles (RPMs) induced autophagy, prevented senescence, and maintained the production of sulphated glycosaminoglycans in human articular chondrocytes isolated from patients with osteoarthritis [44]. In vivo, in mice undergoing surgery to induce osteoarthritis, intra-articular administration of RMPs significantly reduced cartilage damage and inflammation, both when administered preventively and therapeutically, supporting their potential clinical application in slowing the progression of post-traumatic osteoarthritis [44].

Quercetin, a flavonoid with antioxidant and anti-inflammatory properties, shows promising benefits in the treatment of age-related musculoskeletal disorders, playing a key role in the proliferation and differentiation of MSCs [45,46,47,48]. In models of osteoporosis and sarcopenia, quercetin improved BMD, microarchitecture, biomechanics and muscle function by modulating the NF-κB and β-catenin signaling pathways, exerting a targeted action on age-related degenerative processes [49]. Yuan et al. treated ovariectomized female rats with quercetin for 8 weeks and observed an improvement in trabecular microarchitecture and bone biomechanical properties compared to the untreated group. In addition, the authors investigated the effects of quercetin on TNF-α-induced alterations in bone marrow stem cells (BMSCs) osteogenesis, finding an increase in cell viability and mineralization, as well as an increase in the expression of RUNX2, Osx and β-catenin, all factors involved in osteogenic differentiation [50]. Similarly, Zhou and colleagues treated rat BMSCs with quercetin for 7 days, finding an increase in proliferation, ALP activity, calcium deposition, and osteogenic gene expression. Interestingly, quercetin also promoted the expression of angiogenic genes, including vascular endothelial growth factor (VEGF) and angiopoietin-1 (ANG-1), probably through the extracellular signal-regulated kinase (ERK) and p38 signaling pathways, confirming its key role in bone regeneration [51]. On the other hand, the antioxidant properties of quercetin contribute to muscle regeneration, effectively counteracting atrophy processes [52]. The study conducted by Hour and colleagues examined the effect of quercetin on myogenic differentiation in C2C12 murine myoblasts, highlighting its role in cell fusion and migration through the activation of the integrin subunit beta 1 (ITGB1)/focal adhesion kinase (FAK)/paxillin and insulin-like growth factor 1 receptor (IGF-1R)/Akt/mTOR signaling pathways. Importantly, Akt inhibition blocked myogenesis, suggesting that the effect of quercetin depends on this pathway and suggesting its potential use in the prevention of sarcopenia [53].

Among the natural compounds of interest, curcumin stands out for its antioxidant and anti-inflammatory properties, with beneficial effects on bone and muscle tissue. Receno and colleagues observed an increase in muscle mass and contractile function in 32-month-old male rats fed a curcumin diet for 4 months, in association with increased expression of nuclear factor erythroid 2-related factor 2 (NRF2), a key transcription factor in detoxification and antioxidant defense [10]. Similarly, Chen et al. demonstrated a key role for curcumin in the polarization of macrophages towards an anti-inflammatory phenotype, with increased expression of interleukin-4 (IL-4), interleukin-10 (IL-10) and cluster of differentiation 206 (CD206) and reduced expression of interleukin-1 beta (IL-1β), TNF-α, C-C motif chemokine receptor 7 (CCR7), and inducible nitric oxide synthase (iNOS). Interestingly, curcumin also stimulated osteogenic differentiation, osteoblast formation and mineralization in BMSCs, suggesting its therapeutic potential in the treatment of inflammatory diseases and bone regeneration [54].

Vitamin E, a fat-soluble micronutrient composed of tocopherols and tocotrienols, also plays a key role in protecting skeletal muscle from oxidation and cellular damage, improving muscle regeneration and slowing sarcopenia [55]. Several studies have shown that α-tocopherol (ATF) repairs damaged myoblast membranes, while the tocotrienol-rich fraction (TRF) counteracts cellular aging and improves muscle differentiation [56,57]. Khor and colleagues treated primary cultures of human myoblasts with TRF, ATF or N-acetylcysteine (NAC) for 24 h, assessing their effectiveness in restoring oxidative status during replicative senescence [58]. Interestingly, NAC improved cell morphology and reduced senescence, while TRF and ATF reduced ROS production and lipid peroxidation. An increase in SOD activity and reduced/oxidized glutathione (GSH/GSSG) ratio was induced by TRF treatment, suggesting its therapeutic potential against muscle aging [58].

Genistein, an isoflavone belonging to the class of natural phytoestrogens found mainly in soybeans and their derivatives, has been suggested to modulate the senescence of BMSCs, reduce the expression of p53, p16 and p21, and improve mitochondrial function, helping to prevent bone loss [59]. However, the study by Li et al. showed that silencing the ERRα receptor canceled out these effects, suggesting that genistein could be a potential treatment for postmenopausal osteoporosis, with ERRα as a possible therapeutic target. In addition, genistein reduced oxidative damage and promoted the expression of sirtuin 3 (SIRT3) and peroxisome proliferator-activated receptor gamma coactivator 1-alpha (PGC-1α), important regulators of mitochondrial health [14].

Fisetin, a natural flavonol with antioxidant, anti-inflammatory, and senolytic properties, has been proposed for its potential role in promoting health and longevity, although its effects on musculoskeletal regeneration and integrity are still poorly investigated. In this context, Hambright and colleagues treated progeroid Zmpste24^−/−^ mice with fisetin once a week for 4 weeks and observed an improvement in bone microarchitecture associated with a reduction in the expression of p16, interleukin-6 (IL-6) and transforming growth factor beta (TGF-β). Similarly, treatment of MC3T3-E1 pre-osteoblasts and ATDC5 chondrocytes with fisetin for 48 h preserved cell viability and eliminated over 60% of senescent cells, suggesting a potential protective and regenerative effect at the cellular level as well [13]. Interestingly, Zheng and colleagues demonstrated that fisetin exerts anti-inflammatory and chondroprotective effects in osteoarthritis through the activation of SIRT1 [60]. In chondrocytes stimulated with IL-1β, fisetin reduced the expression of pro-inflammatory mediators, such as nitric oxide (NO), prostaglandin E2 (PGE2), TNF-α and IL-6, and degradative enzymes, such as cyclooxygenase-2 (COX-2), iNOS, matrix metalloproteinase-3 (MMP-3), matrix metalloproteinase-13 (MMP-13) and A disintegrin and metalloproteinase with thrombospondin motifs 5 (ADAMTS-5). These effects were reversed by inhibition of SIRT1 with sirtinol, confirming its central role. In vivo, fisetin administration attenuated joint degeneration in mice with induced osteoarthritis, as well as reducing subchondral bone plaque thickness and alleviating synovitis, confirming its efficacy in modulating disease progression through SIRT1 [60].

Finally, Ramirez-Sanchez and colleagues highlighted the potential of epicatechin, an epimer of cocoa flavonol, in counteracting age-related muscle atrophy [61]. Specifically, treatment of 23-month-old male Sprague Dawley rats with 1 mg/kg/day oral epicatechin for 8 weeks promoted a significant improvement in strength, endurance, and muscle mass. In addition, epicatechin increased insulin-like growth factor 1 (IGF-1) levels and activated the Akt/mTORC1 anabolic pathway, reducing pro-inflammatory TNF-α blood levels and NF-κB signaling. Interestingly, the treatment inhibited muscle degradation markers such as muscle ring-finger protein-1 (MuRF1) and Atrogin-1, and modulated muscle growth regulators with a reduction in myostatin and an increase in follistatin. Importantly, downregulation of the myostatin pathway resulted in a significant reduction in phosphorylated suppressor of mothers against decapentaplegic homolog (SMAD) 2/3 protein levels, highlighting the ability of epicatechin to counteract myostatin signaling-associated muscle atrophy. These results suggest that epicatechin exerts a protective effect on skeletal muscle during aging, supporting its potential in preventing sarcopenia [61].

**Table 1:** A schematic representation of the natural compounds investigated, the molecular targets involved, and the biological effects observed.

Natural Compound	Chemical Class	Molecular Target	Study Population	Evidence	Reference
**Resveratrol**	Polyphenolic phytoalexin	SIRT1/AMPK	MSCs isolated from 12-month-old Kunming mice treated with resveratrol for 48 h	- Reduced ROS - Improved cell viability - Promoted extracellular matrix calcification - Increased expression of ALP, OCN, OPN, Col-I, RUNX2	[36]
		SIRT1/FoxO1	- 8-week-old ovariectomized female mice treated with resveratrol intraperitoneally (40 mg/kg body weight) once daily for 8 weeks - Murine osteoblasts subjected to H_2_O_2_-induced oxidative stress, treated with 0.1 mM resveratrol for 1, 3, and 5 days	In vivo: - Improved bone microarchitecture - Increased expression of ALP, RUNX2, Osx In vitro: - Promoted cell proliferation - Increased ALP activity - Increased SOD expression - Reduced ROS	[12]
		SIRT1	25-month-old Sprague-Dawley rats treated with oral resveratrol (150 mg/kg/day) for 6 weeks	- Increased AMPK, SIRT1, and Bcl-2 - Reduced Bax and p53	[37]
**Rapamycin**	Macrocyclic lactone	mTOR	30-month-old female HET3 mice treated with rapamycin intraperitoneally (6 mg/kg body weight) every other day for 8 weeks	- mTORC1 inhibition - Reduced GDF15 and STAT3 expression - Attenuation of oxidative stress - Reduced muscle atrophy	[42]
			15 wild-type female mice aged 8 weeks treated with 4 mg/kg rapamycin intraperitoneally every other day for 12 weeks	- Reduced femoral bone mineral content - Reduced BMD - Increased serum CTX-1 - Increased bone resorption	[43]
			- Chondrocytes isolated from osteoarthritic patients treated with 1 µM RPMs for 48 h - Wild-type C57BL/6 male mice with post-traumatic osteoarthritis treated with 1.8 µg RPMs by intramuscular injection on days 7, 24 and 42 in the prophylactic study, and on days 24 and 42 in the therapeutic study	In vitro: - Autophagy induction - Reduced cellular senescence - Maintenance of sulphated glycosaminoglycan production In vivo: - Reduction in cartilage damage and inflammation in both preventive and therapeutic phases	[44]
**Quercetin**	Flavonoid	NF-κB/β-catenin	- 8–10-week-old ovariectomized female Sprague Dawley rats treated with 50 mg/kg quercetin per day for 8 weeks - BMSCs treated with 1 µM quercetin for 24 h after stimulation with 5 ng/mL TNF-α	In vivo: - Improved trabecular microarchitecture and bone biomechanical properties In vitro: - Increased cell viability - Promoted bone mineralization - Increased RUNX2, Osx and β-catenin expression	[50]
		ERK/p38	BMSCs isolated from 4-week-old male rats treated with quercetin at concentrations of 0, 1, 2, 5, and 10 μM for 7 days	Optimal results at 2 μM concentration: - Increased cell proliferation - Increased ALP activity - Promoted calcium deposition and osteogenesis - Increased VEGF and ANG-1 expression	[51]
		ITGB1/FAK/paxillin, IGF-1R/Akt/mTOR	C2C12 mouse myoblasts treated with quercetin at concentrations of 0, 2.5, 12.5, 25 and 50 µM for 7 days every 48 h	Optimal results at 12.5 µM concentration: - Increased cell fusion and migration - Increased myogenic differentiation - Promoted muscle regeneration	[53]
**Curcumin**	Polyphenol	NRF2	18 male F344xBN rats aged 32 months fed with 0.2% curcumin for 4 months	- Increased muscle mass and contractile function - Increased NRF2 expression - Reduced oxidative damage	[10]
**Vitamin E**	Tocopherols/Tocotrienols	NF-kB/STAT3	Human myoblasts treated with 10 µM TRF, 10 µM ATF, or 5 µM NAC for 24 h	- Improved cell morphology and reduced senescence after NAC treatment - Reduced ROS production and lipid peroxidation after RTF and ATF treatments - Increased SOD activity and GSH/GSSG ratio after TRF treatment	[58]
**Genistein**	Isoflavone	ERRα	BMSCs isolated from 12-week-old ovariectomized female Sprague-Dawley rats treated with genistein at doses of 1 μM and 10^−2^ μM for 3 days	- Reduced p53, p16 and p21 expression - Improved mitochondrial function - Reduced oxidative damage - Increased SIRT3 and PGC-1α expression - Reduced bone loss	[14]
**Fisetin**	Flavonol	SIRT1	- Primary human chondrocytes pretreated with fisetin at doses of 1, 5, 10 μM for 2 h, followed by stimulation with or without 10 ng/mL IL-1β for 24 h - 10-week-old male wild-type C57BL/6 mice treated with 20 mg/kg fisetin orally every day for 8 weeks	In vitro: - Reduced expression of NO, PGE2, TNF-α, IL-6, COX-2, iNOS, MMP-3, MMP-13 and ADAMTS-5 - Greater increase in SIRT1 expression at a concentration of 10 μM fisetin In vivo: - Attenuation of joint degeneration - Reduced subchondral bone plaque thickness - Reduced severity of synovitis	[60]
**Epicatechin**	Flavonol	Akt/mTOR	23-month-old male Sprague Dawley rats treated with 1 mg/kg/day oral epicatechin for 8 weeks	- Improved strength, endurance, and muscle mass - Increased IGF-1 and follistatin - Reduced TNF-α, myostatin - Inhibited NF-κB signaling, MuRF1 and Atrogin-1	[61]

SIRT1: sirtuin 1; AMPK: AMP-activated protein kinase; MSCs: mesenchymal stem cells; ROS: reactive oxygen species; ALP: alkaline phosphatase; OCN: osteocalcin; OPN: osteopontin; Col-I: type I collagen; RUNX2: runt-related transcription factor 2; FoxO1: forkhead box O1; Osx: osterix; SOD: superoxide dismutase; Bcl-2: B-cell lymphoma 2; Bax: Bcl-2-associated X protein; mTOR: mechanistic target of rapamycin; mTORC1: mTOR complex 1; GDF15: growth differentiation factor 15; STAT3: signal transduced and activator of transcription 3; BMD: bone mineral density; CTX-1: C-terminal telopeptide of type 1 collagen; RPMs: rapamycin-loaded poly(lactic-co-glycolic acid) microparticles; NF-κB: nuclear factor kappa-light-chain-enhancer of activated B cells; BMSCs: bone marrow stem cell; TNF-α: tumor necrosis factor-alfa; ERK: extracellular signal-regulated kinase; VEGF: vascular endothelial growth factor; ANG-1: angiopoietin-1; ITGB1: integrin subunit beta 1; FAK: focal adhesion kinase; IGF-1R: insulin-like growth factor 1 receptor; NRF2: nuclear factor erythroid 2-related factor 2; TRF: tocotrienol-rich fraction; ATF: α-tocopherol; NAC: N-acetylcysteine; GSH/GSSG: reduced/oxidized glutathione; ERRα: estrogen-related receptor alpha; SIRT3: sirtuin 3; PGC-1α: peroxisome proliferator-activated receptor gamma coactivator 1-alpha; IL-1β: interleukin-1 beta; NO: nitric oxide; PGE2: prostaglandin E2; IL-6: interleukin-6; COX-2: cyclooxygenase-2; iNOS: inducible nitric oxide synthase; MMP-3: matrix metalloproteinase-3; MMP-13: matrix metalloproteinase-13; ADAMTS-5: A disintegrin and metalloproteinase with thrombospondin motifs 5; IGF-1: insulin-like growth factor 1; MuRF1: muscle ring-finger protein-1.

## 4. Synthetic Compounds

**Figure 2:**
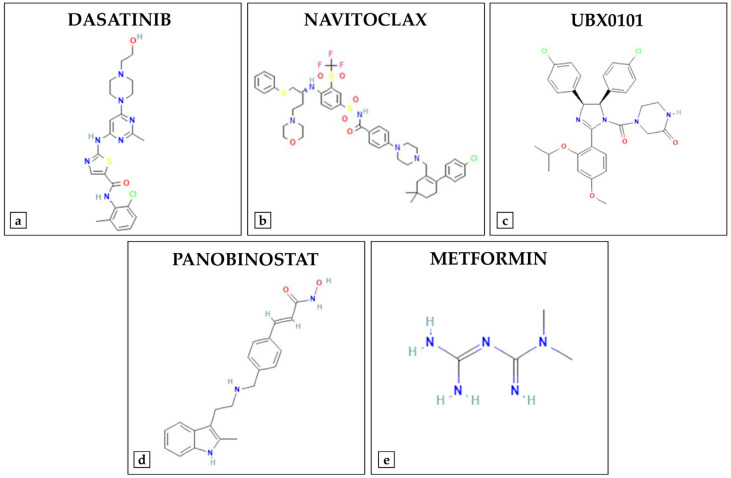
Chemical structure of the main synthetic compounds with senotherapeutic properties. (**a**) Dasatinib. (**b**) Navitoclax. (**c**) UBX0101. (**d**) Panobinostat. (**e**) Metformin. Images acquisition from PubChem.

In recent years, numerous synthetic compounds have been investigated for the treatment of cellular senescence in musculoskeletal diseases, with a particular focus on senolytic agents and metabolic regulators (Figure 2) (Table 2).

Among the most promising approaches, the combination of dasatinib and quercetin has been shown to be effective in selectively eliminating senescent cells and reducing the secretion of pro-inflammatory factors in various tissues [62]. Particularly, dasatinib is a tyrosine kinase inhibitor which, when combined with quercetin, inhibits the phosphoinositide 3-kinase (PI3K)/Akt and Bcl-2/B-cell lymphoma-extra large (Bcl-xL) signaling pathways, promoting the removal of dysfunctional cells [63]. Wang and colleagues demonstrated that treatment with dasatinib and quercetin for 2 and 4 months is effective in preventing bone loss in ovariectomized rats, reducing the accumulation of senescent cells and restoring the functionality of MSCs [6]. Notably, these effects were associated with reduced expression of NF-κB, a key transcription factor in the regulation of inflammatory response and cellular senescence. Furthermore, local implantation of a hydrogel containing dasatinib and quercetin combined with bone morphogenetic protein 2 (BMP-2) showed a synergistic effect in bone regeneration, highlighting its therapeutic potential in stimulating osteogenesis [6]. More recently, Zhao and colleagues demonstrated that treatment with dasatinib and quercetin for 10 weeks improved joint degeneration in a model of facet joint osteoarthritis, a condition characterized by activation of the NF-κB pathway, as well as reducing the expression of senescence markers, including p16, p21 and p53, and pro-inflammatory molecules, such as IL-1β and IL-6. Treatment with dasatinib and quercetin also reduced SASP release in primary chondrocyte cultures, slowing tissue damage and the progression of osteoarthritis [64].

On the other hand, navitoclax, an inhibitor of the Bcl-2/Bcl-xL signaling pathway, initially developed as an anti-tumor agent and subsequently studied to assess its effects on bone mass and osteoprogenitor cells in elderly mouse models, did not show favorable results. Sharma et al. observed significant loss of trabecular bone tissue and impaired BMSCs function in aged mice treated with navitoclax for 2 weeks, in association with reduced mineralized matrix production and decreased expression of the osteoprogenitor gene Osx [7]. In vitro, treatment of BMSCs with navitoclax for 5 days reduced the senescent cell burden, but compromised bone formation and increased cytotoxicity. Therefore, its use in age-related skeletal diseases may be limited due to its negative effects on bone regeneration and osteoprogenitor function [7].

Similarly, the study by Chin and colleagues examined the effects of treatment with UBX0101, a p53/p21 pathway inhibitor, in a mouse model of post-traumatic osteoarthritis, observing a reduction in oxidized proteins in cartilage and synovial fluid. However, different responses to treatment were observed between young 10-week-old mice and older 19-month-old mice, highlighting the need to develop therapies tailored to different age groups [8].

Interestingly, alterations in articular cartilage have been associated with the suppression of FoxO transcription factors, as mice lacking FoxO1 or FoxO3 show early-onset osteoarthritis. Furthermore, FoxO1 is known to reduce oxidative stress in chondrocytes and increase the expression of autophagic genes, suggesting a protective role in the context of osteoarthritis [65]. In this regard, Ohzono et al. treated primary murine chondrocytes and synoviocytes previously stimulated with IL-1β to induce inflammation with panobinostat, a pan-specific histone deacetylase inhibitor [15]. The treatment increased the expression of joint function genes, including FoxO1, proteoglycan 4 (PRG4) and aggrecan (ACAN), as well as reducing the expression of nitric oxide synthase 2 (NOS2), an inducible nitric oxide synthase gene involved in the inflammatory response. In vivo, treatment with panobinostat attenuated histopathological alterations in cartilage, synovium and subchondral bone, resulting in an improvement in pain-related behavioral parameters. Therefore, panobinostat has been proposed as a promising senomorph capable of mitigating joint aging by reactivating the FoxO signaling pathway and counteracting inflammation and tissue degradation [15].

Finally, metformin, a well-known calorie restriction mimetic, exerts its anti-aging effects in musculoskeletal diseases through the activation of AMPK and the inhibition of mTOR, promoting cellular autophagy and attenuating oxidative stress [66]. Mai et al. treated mouse osteoblasts and the MC3T3-E1 cell line with different concentrations of metformin, observing a dose-dependent increase in osteoprotegerin (OPG) expression and a reduction in receptor activator of nuclear factor kappa-B ligand (RANKL) expression at both the protein and mRNA levels. In vivo, oral administration of metformin to ovariectomized rats for 2 months also promoted an increase in BMD and prevented bone loss, confirming its protective role against osteoporosis [67]. In addition, metformin is known to activate the AMPK/SIRT1 signaling pathway, which plays a key role in the treatment of osteoarthritis. In this regard, Wang et al. demonstrated that metformin treatment protects against IL-1β-induced extracellular matrix degradation in both chondrocytes and mouse models of osteoarthritis by increasing autophagy, improving mitochondrial function and reducing ROS accumulation [68]. Interestingly, metformin has also shown cytoprotective properties against sarcopenia, promoting the restoration of autophagic flux and improving mitochondrial function in models of cellular aging. Bang and colleagues treated late-passage C2C12 cells with metformin at two concentrations for 2 days, observing a reduction in cellular senescence, improved autophagy, and stimulation of myogenic differentiation only at the low dose of 75 µM. In contrast, administration of metformin at a higher dosage, 500 µM, exacerbated muscle dysfunction and impaired cellular health, suggesting a dose-dependent effect of metformin on muscle quality and function [16].

**Table 2:** A schematic representation of the synthetic compounds investigated, the molecular targets involved, and the biological effects observed.

Synthetic Compound	Molecular Target	Study Population	Evidence	Reference
**Dasatinib**	NF-κB	12-month-old female ovariectomized rats treated orally with 5 mg/kg dasatinib and 50 mg/kg quercetin for 2 and 4 months	- Reduced bone loss - Reduced senescent cell accumulation - Restoration of MSCs functionality - Reduced NF-kB expression	[6]
		- Male C57BL/6 mice with facet joint osteoarthritis aged between 8 and 10 weeks treated orally with 5 mg/kg dasatinib and 50 mg/kg quercetin for 10 weeks - Primary mouse chondrocytes treated with 200 nM dasatinib and 10 μm quercetin for 48 h	In vivo: - Improvement in joint degeneration - Reduced expression of p16, p21, p53, IL-1β, IL-6 In vitro: - Attenuation of senescence and release of SASPs	[64]
**Navitoclax**	Bcl-2/Bcl-xL	- 20 male (*n* = 10) and female (*n* = 10) 24-month-old C57BL/6 mice treated orally with 50 mg/kg navitoclax once daily for 2 weeks - Mouse BMSCs treated with 5 μM navitoclax for 5 days	In vivo: - Trabecular bone loss - Reduced mineralized matrix production - Reduced Osx expression In vitro: - Reduced cellular senescence - Compromised bone formation - Increased cytotoxicity	[7]
**UBX0101**	p53/p21	10-week-old or 19-month-old male C57BL/6 mice with induced osteoarthritis treated with 1 mM UBX0101 via six intra-articular injections every two days	Reduction in the presence of oxidized proteins in cartilage and synovial fluid in 19-month-old mice	[8]
**Panobinostat**	FoxO	- Primary murine chondrocytes and synoviocytes treated with panobinostat at doses of 10, 50, and 100 nM for 24 h after stimulation with 5 ng/mL IL-1β - C57BL/6J mice treated with panobinostat at doses of 100 μg/kg and 2.5 mg/kg intraperitoneally 3 times a week for 11 weeks	In vitro: - Increased FoxO1, PRG4, ACAN expression - Reduced NOS2 expression In vivo: - Attenuated histopathological alterations in cartilage, synovium and subchondral bone - Improved pain-related behavioral parameters	[15]
**Metformin**	AMPK/SIRT1	- Mouse osteoblasts and MC3T3-E1 cell line treated with metformin at concentrations of 0–800 µM for 0–72 h - 45 female Sprague–Dawley rats aged 8–10 weeks, ovariectomized and treated with 100 mg/kg metformin orally every day for two months	In vitro: - Stimulated OPG and reduced RANKL mRNA and protein expression In vivo: - Increased BMD - Prevented bone loss - Increased OPG expression - Decreased RANKL expression	[67]
		- 15 male C57BL/6 mice aged 8 weeks treated with 16.5 mg/mL metformin intra-articularly every three days for 8 weeks - Chondrocytes isolated from 6 to 8-week-old male C57BL/6 mice treated with 1 mM metformin administered one hour before stimulation with 10 ng/mL IL-1β	In vivo: - Restored overexpression of MMP-13 and underexpression of Col-II in articular cartilage In vitro: - Increased phosphorylation of AMPK and SIRT1 - Promoted autophagy - Reduced catabolism and apoptosis	[68]
		C2C12 myoblasts at advanced passages treated with metformin at doses of 75 µM and 500 µM every 24 h for 2 days	Optimal results at 75 µM dose: - Reduced cellular senescence - Improved autophagy - Promoted myogenic differentiation	[16]

NF-κB: nuclear factor kappa-light-chain-enhancer of activated B cells; MSCs: mesenchymal stem cells; IL-1β: interleukin-1 beta; IL-6: interleukin-6; SASPs: senescence-associated secretory phenotypes; Bcl-2: B-cell lymphoma 2; Bcl-xL: B-cell lymphoma-extra large; BMSCs: bone marrow stem cell; Osx: osterix; FoxO: forkhead box O; FoxO1: forkhead box O1; PRG4: proteoglycan 4; ACAN: aggrecan; NOS2: nitric oxide synthase; RANKL: receptor activator of nuclear factor kappa-B ligand; OPG: osteoprotegerin; BMD: bone mineral density; AMPK: AMP-activated protein kinase; SIRT1: sirtuin 1; MMP-13: matrix metalloproteinase-13; Col-II: type II collagen.

## 5. Physical Exercise as a Non-Pharmacological Intervention Against Musculoskeletal Aging

Physical exercise is a valuable tool in the prevention and treatment of age-related musculoskeletal disorders, as it is known to reduce oxidative stress, improve mitochondrial function and modulate the signaling pathways involved in energy metabolism and inflammatory response [69,70]. In addition, physical exercise helps maintain the balance between the proliferation and elimination of senescent cells, thus delaying tissue deterioration and contributing to healthy aging [71]. In this context, several studies have shown that aerobic and resistance exercise can reduce the expression of cellular senescence markers, improving quality of life and increasing longevity (Table 3) [72].

The AMPK signaling pathway and the nicotinamide adenine dinucleotide (NAD^+^)/reduced NAD^+^ (NADH) ratio, both crucial in controlling energy homeostasis and regulating cell survival and reprogramming mechanisms, are among the main molecular targets involved in physiological adaptations to exercise [73,74]. Yoon and colleagues subjected 19-month-old mice to 4 weeks of treadmill running, 30 min a day for 5 days a week, observing a significant reduction in markers of cellular senescence, including p16 and p21, compared to the sedentary group. Interestingly, pretreatment of C2C12 myoblasts with AICAR, a pharmacological AMPK agonist, prevented the doxorubicin-induced senescence phenotype, suggesting that AMPK activation is a promising strategy for counteracting muscle aging and preserving tissue health [24]. However, further preclinical studies are needed to evaluate the efficacy and safety of AICAR, as some evidence has raised concerns regarding its pharmacokinetics, including low bioavailability when administered orally and a short half-life when administered intravenously [75]. In agreement, Fan and colleagues investigated whether long-term exercise combined with spermidine, a natural polyamine, could inhibit apoptosis and activate autophagy in a rat model with muscle atrophy induced by D-galactose (D-gal) treatment [76]. Specifically, 4-month-old rats were divided into a control group (Con), a D-gal-treated group (D), a D-gal- and spermidine-treated group (DS), a D-gal- and exercise-treated group (DE), and a D-gal-, spermidine-, and exercise-treated group (DES). The DE and DES groups underwent 60 min of swimming training per day, 5 days per week for 6 weeks. Not surprisingly, the DES group showed a significant reduction in senescence-associated β-galactosidase (SA-β-gal) compared to the other experimental groups, in association with increased SOD activity and reduced malondialdehyde (MDA) levels. Importantly, the combined treatment promoted autophagy by activating the signaling pathway AMPK/FoxO3a [76].

Physical exercise is known to modulate the expression of SIRT1, an NAD^+^-dependent deacetylase, interacting with transcriptional factors essential for cell survival, such as PGC-1α, FoxO, NF-κB and p53, contributing to the reduction in oxidative stress and counteracting cell senescence [77,78,79]. Interestingly, PGC-1α appears to exert its effects by interacting directly with ERRα, contributing to the regulation of genes involved in mitochondrial biogenesis and fatty acid oxidation [80]. In this regard, recent evidence has shown that SLU-PP-332, an exercise mimetic, is able to increase the expression of ERRα, confirming the central role of the PGC-1α/ERRα axis in the beneficial effects of exercise at the cellular level [81,82]. Huang and colleagues highlighted how a swimming training program of 40 min a day, 5 days a week for 12 weeks modulates the expression of proteins involved in the SIRT1/PGC-1α signaling pathway in female rats of different ages. Particularly, SIRT1, PGC-1α and AMPK levels were upregulated in the gastrocnemius and soleus muscles of all trained animals compared to sedentary groups, confirming the key role of exercise in modulating the metabolic pathways that regulate muscle function and efficiency [83]. Koltai et al. subjected 3- and 26-month-old rats to 30 min of treadmill running per day for 6 weeks, finding an increase in SIRT1 activity and a restoration of NAD^+^ and nicotinamide phosphoribosyltransferase (NAMPT) levels in both trained groups. In addition, exercise increased uncoupling protein 3 (UCP3) expression and reduced hypoxia-inducible factor 1-alpha (HIF-1α) and VEGF levels, counteracting oxidative stress and age-related mitochondrial damage [84]. More recently, Cariati et al. investigated the effects of vibratory training on bone tissue in young, adult and elderly mice, exposing them to a whole body vibration (WBV) protocol consisting of 3 series of 2 min and 30 s, with a recovery period of the same duration, 3 times a week for 12 weeks [85]. Their results showed an increase in bone volume and trabecular thickness in all trained groups, in association with an increase in the expression of SIRT1 and fibronectin type III domain-containing protein 5 (FNDC5), a precursor of the hormone irisin [86]. In addition, vibratory training significantly reduced the expression of NADPH oxidase 4 (NOX4), a known inducer of oxidative stress, confirming itself as a valid alternative for counteracting musculoskeletal decline in elderly and/or sedentary subjects [85].

SIRT3, which is mainly expressed in mitochondria, also plays a key role in regulating energy metabolism and mitochondrial biogenesis [87,88]. Recent evidence has shown a close association between aging and a reduction in the number of mitochondria in the dendritic processes of osteocytes, suggesting that SIRT3 deficiency is a determining factor in the inhibition of osteoblastic differentiation and the loss of the beneficial effects of exercise on skeletal muscle [89,90]. In this regard, the randomized controlled study by Johnson et al. examined the impact of 8 weeks of cycling at 65% of peak oxygen consumption for 60 min on the skeletal muscle of 20 elderly individuals, observing a significant increase in the content of mitochondrial deacetylase SIRT3 compared to sedentary subjects. In addition, exercise promoted an increase in catalase (CAT) and superoxide dismutase 2 (SOD2), confirming its effectiveness in promoting mitochondrial adaptation and enhancing the antioxidant capacity of muscle, helping to counteract the effects of aging [91].

Notably, weight-bearing exercises such as running, jumping, and weightlifting induce mechanical stress that stimulates bone formation by activating osteocytes, promoting the release of growth factors, and guiding the differentiation of mesenchymal stem cells into osteoblasts [92]. Although the underlying molecular mechanisms are largely unknown, irisin and β-aminoisobutyric acid (BAIBA), two myokines released by skeletal muscle in response to exercise, appear to directly slow age-related osteocyte senescence by preserving mitochondrial function and the processes of biogenesis and mitophagy [93,94]. In this context, physical exercise is known to regulate the PI3K/Akt signaling pathway, which plays a key role in muscle regeneration, mainly through the action of growth factors such as IGF-1, brain-derived neurotrophic factor (BDNF) and epidermal growth factor (EGF). Particularly, IGF-1 is produced locally in the muscle and activates the PI3K/Akt signaling pathway, stimulating the proliferation and differentiation of myoblasts [95]. The resulting activation of mTORC1 promotes protein synthesis and muscle growth, playing a crucial role in age-related diseases [96,97]. Bae and colleagues subjected 8-week-old male rats to treadmill running for 60 min a day, 4 times a week for 8 weeks, observing an increase in the phosphorylation of Akt, FoxO3a and PGC-1α in the gastrocnemius and soleus muscles compared to the sedentary group, as well as a significant reduction in the levels of MuRF1 and Atrogin-1, which are involved in muscle atrophy [98]. On the other hand, the study by Zeng and colleagues showed that exposing elderly rats to resistance exercise, which consisted of climbing an inclined ladder with progressive loads applied to their tails until reaching 80% of their body weight, improved muscle mass and fiber structure and counteracted atrophy [99]. Interestingly, exercise reduced the expression of Atrogin-1 and MuRF1 and stimulated autophagy through an increase in Beclin1 and Bcl-2, an improvement in the microtubule-associated protein 1A/1B-light chain 3 (LC3) II/LC3-I ratio, and a reduction in p62 and Bax levels. In addition, the authors observed an improvement in mitochondrial function due to increased levels of PGC-1α, mitofusin 2 (Mfn2), dynamin-related protein 1 (Drp1) and PTEN-induced kinase 1 (PINK1), confirming it as a valid strategy for counteracting sarcopenia and preserving muscle health during aging [99].

Finally, the study by Yan and colleagues identified the transcription factor NRF2 as a key element in the benefits of exercise on aging skeletal muscle [100]. In 22-month-old wild-type mice, exercise led to improved muscle function and a reversal of the sarcopenic phenotype, associated with a significant increase in NRF2 expression. These effects were greatly attenuated in NRF2 knockout mice of the same age, highlighting the crucial role of this factor in the adaptive response to exercise in old age. At the cellular level, Nrf2 appears to promote the stabilization of the Drp1, promoting mitochondrial fission and contributing to the maintenance of mitochondrial homeostasis [101]. In this regard, the same authors demonstrated that pharmacological activation of NRF2 via sulforaphane reproduced similar effects in senescent C2C12 muscle cells, confirming its potential as a therapeutic target in sarcopenia [100].

**Table 3:** A schematic representation of the molecular targets modulated by physical exercise and their biological effects.

Molecular Target	Study Population	Evidence	Reference
**AMPK**	- 9-week-old and 19-month-old male C57/BL6 mice subjected to moderate treadmill running (8 m/min) for 2 days, followed by 4 weeks of exercise training, 30 min twice a day with 1 h of rest, 5 days/week - Differentiated C2C12 myoblasts pretreated with 100 μM AICAR for 30 min prior to administration of 1 μM doxorubicin for 15 min	In vivo: - Reduced cellular senescence and p16 and p21 expression in 19-month-old male In vitro: - Pretreatment with AICAR attenuates doxorubicin-induced AMPK phosphorylation and prevents the cellular senescence phenotype	[24]
**AMPK/FOXO3a**	4-month-old male Sprague-Dawley rats: - Group D: treated with D-gal (200 mg/kg/day) - Group DS: D-gal + spermidine (5 mg/kg/day) - Group DE: D-gal + exercise (60 min of swimming per day, 5 days per week, for 6 weeks) - Group DES: D-gal + exercise + spermidine	Optimal results in the DES group: - Reduced SA-β-gal - Increased SOD activity - Reduced MDA levels - Promoted autophagy	[76]
**SIRT1/PGC-1α**	3-, 12- and 18-month-old female Sprague-Dawley rats subjected to swimming training for 40 min/day, 5 days/week for 12 weeks	- Upregulation of SIRT1, PGC-1α, and AMPK in the gastrocnemius and soleus muscles - Improved muscle function	[83]
**SIRT1**	Male Wistar rats aged 3 and 26 months subjected to treadmill running for 2 weeks at a speed of 10 m/min and a 5% incline for 30 min, progressively increased to 60% of VO2max	- Increased SIRT1 activity - Restored NAD^+^ and NAMPT levels - Increased UCP3 expression - Reduced HIF-1α and VEGF expression - Reduced oxidative stress and mitochondrial damage	[84]
**NOX4/SIRT1/FNDC5**	Wild-type BALB/c male mice aged 4, 12 and 24 months subjected to a WBV protocol consisting of 3 series of 2 min and 30 s each, with 2 min and 30 s of recovery, 3 days a week for 12 weeks	- Increase bone volume and trabecular thickness - Up-regulation of SIRT1 and FNDC5 - Down-regulation of NOX4	[85]
**SIRT3**	23 young subjects (aged 18–30, 11 women and 12 men) and 20 elderly subjects (aged ≥65, 9 women and 11 men) underwent 8 weeks of cycling at 65% of peak oxygen consumption for 60 min	- Reduction in oxidative damage in the skeletal muscle of young subjects - Increased SIRT3, CAT and SOD2 in the skeletal muscle of elderly subjects	[91]
**Akt/PGC-1α/FoxO3a**	7-week-old male Wistar rats subjected to treadmill running for 60 min a day, 4 times a week for 8 weeks	- Increase in the phosphorylation of Akt, FoxO3a and PGC-1α in the gastrocnemius and soleus muscles - Reduced MuRF1 and Atrogin-1 expression - Attenuation of muscle atrophy	[98]
**Akt/mTOR, Akt/FoxO3a**	21-month-old male Wistar rats subjected to resistance exercise, which consisted of climbing an inclined ladder with progressive loads applied to their tails until reaching 80% of their body weight	- Reduced Atrogin-1, MuRF1, p62 and Bax expression - Promoted autophagy - Increased expression of Beclin1, Bcl-2 and LC3-II/LC3-I ratio - Improved mitochondrial function - Increased PGC-1α, Mfn2, Drp1 and PINK1 expression	[99]
**NRF2**	22-month-old wild-type C57/BL6/J male mice and 22-month-old NRF2 knockout mice subjected to 20 min of treadmill running per day for 8 weeks, with 1 day of rest per week Senescent C2C12 myoblasts treated with sulforaphane at different concentrations (0, 1, 2, 3, 4, 5 and 6 μM) for 20 h	In vivo (wild-type mice): - Improved muscle function - Reverted sarcopenic phenotype - Increased NRF2 expression In vitro: - Increased NRF2 and Drp1 expression - Promoted mitochondrial fission - Reduced cellular senescence	[100]

AMPK: AMP-activated protein kinase; AICAR: 5-aminoimidazole-4-carboxamide ribonucleotide; FOXO3a: forkhead box O3a; SA-β-gal: senescence-associated β-galactosidase; SOD: superoxide dismutase; MDA: malondialdehyde; SIRT1: sirtuin 1; PGC-1α: peroxisome proliferator-activated receptor gamma coactivator 1-alpha; NAD^+^: nicotinamide adenine dinucleotide; NAMPT: nicotinamide phosphoribosyltransferase; UCP3: uncoupling protein 3; HIF-1α: hypoxia-inducible factor 1-alpha; VEGF: vascular endothelial growth factor; NOX4: NADPH oxidase 4; FNDC5: fibronectin type III domain-containing protein 5; WBV: whole body vibration; SIRT3: sirtuin 3; CAT: catalase; SOD2: superoxide dismutase 2; MuRF1: muscle ring-finger protein-1; mTOR: mechanistic target of rapamycin; Bcl-2: B-cell lymphoma 2; LC3-II/LC3-I: microtubule-associated protein 1A/1B-light chain 3 II/microtubule-associated protein 1A/1B-light chain 3 I; Mfn2: mitofusin 2; Drp1: dynamin-related protein 1; PINK1: PTEN-induced kinase 1; NRF2: nuclear factor erythroid 2-related factor 2.

## 6. Clinical Evidence on the Efficacy and Safety of Senotherapeutics and/or Combined with Physical Exercise

Although anti-senescence compounds have shown promising beneficial effects in the treatment of musculoskeletal disorders, most of the available evidence comes from preclinical studies. In fact, current knowledge on the efficacy and safety of these molecules in humans is still limited (Table 4), making further studies necessary to evaluate their real therapeutic potential and ensure their safe use in clinical practice.

In this context, Alway et al. conducted a double-blind randomized controlled trial (RCT) on 12 men and 18 women aged between 65 and 80 who underwent 12 weeks of exercise and were treated with 500 mg/day resveratrol, observing an improvement in mitochondrial density and muscle fatigue resistance induced by the combination therapy compared to exercise alone. In addition, a significant increase in fiber area and number of myonuclei in the vastus lateralis muscle was detected, suggesting combined therapy as an optimal approach to reversing muscle decline in sarcopenic elderly subjects [102]. Similarly, Harper and colleagues conducted a three-arm, two-site pilot RCT involving 60 adults ≥ 65 years with functional limitations to assess the safety and feasibility of combining physical exercise with resveratrol at two different doses, 500 mg/day (EX500) and 1000 mg/day (EX1000) [103]. The exercise protocol consisted of two weekly sessions for 12 weeks, including walking and whole-body resistance training. Safety and feasibility were evaluated through the occurrence of adverse events and adherence to the protocol, respectively. Overall, the authors reported 27 adverse events, primarily related to gastrointestinal or musculoskeletal issues, with 12 occurring in the EX500 group and 7 in the EX1000 group. Adherence to the exercise sessions was 82%, while adherence to resveratrol supplementation was 85%. Improvements in physical function, assessed via walking endurance, were observed across all groups, with a clinically meaningful increase from baseline to week 12 in the EX1000 group. Interestingly, the authors suggested that combining exercise with resveratrol supplementation may enhance aerobic capacity by improving skeletal muscle mitochondrial function, as indicated by an increase in citrate synthase activity, a well-established marker of mitochondrial volume. However, further studies are needed to better characterize the muscle-specific responses to different resveratrol dosages [103]. Unfortunately, there are currently no RCTs available to determine the effectiveness of combined exercise and resveratrol treatment in the context of bone health. However, Wong and colleagues conducted a double-blind, crossover RCT of 128 postmenopausal women ≥ 65 years treated with 75 mg/day resveratrol for 12 months [104]. Importantly, a 1.3% increase in lumbar spine BMD was observed, as well as an increase in femoral neck and total hip BMD, and a reduction in plasma CTX-1, suggesting a reduction in the risk of osteoporotic fracture at 10 years. In addition, itching, menses, prolapsed bladder, pre-scheduled eye operation were reported among the most frequent adverse events in the treated group [104]. These results are consistent with those of other clinical studies evaluating the benefits of resveratrol supplementation on bone health in other target populations, confirming its safety, efficacy, and clinical relevance [105]. Among these, Hussain et al. conducted a double-blind RCT to evaluate the efficacy of resveratrol as an adjuvant to meloxicam on pain and functional activity in 92 patients with knee osteoarthritis [106]. Supplementation with 500 mg/day resveratrol for 90 days significantly improved pain symptoms and physical function compared to the placebo group, highlighting both its efficacy and tolerability in combination with non-steroidal anti-inflammatory drugs (NSAIDs). Although no serious adverse events were reported in the treated group, one of the main critical issues associated with the use of resveratrol is its low bioavailability [106]. This pharmacokinetic limitation suggests the need to develop innovative strategies aimed at improving its absorption, stability, and, consequently, therapeutic efficacy [107].

Regarding the beneficial effects of rapamycin, Moel et al. conducted a double-blind RCT to evaluate its long-term safety in 114 healthy subjects aged 50 to 85 years treated with placebo, 5 or 10 mg of oral rapamycin once a week for 48 weeks [108]. Adverse events were similar between groups, although gastrointestinal symptoms were more frequent in participants treated with rapamycin. In addition, the group treated with 10 mg of rapamycin showed significant improvements in lean mass and self-reported pain, while an improvement in self-reported emotional well-being and overall health was recorded with the lower dosage. Unfortunately, no significant improvements were observed in visceral adipose tissue, bone mineral content, and BMD, suggesting the need for further studies to verify the benefits of rapamycin in the context of musculoskeletal aging [108]. Interestingly, Kraig and colleagues investigated the efficacy of oral rapamycin administration in 25 healthy elderly subjects aged between 70 and 95 years [109]. Specifically, the study included a phase 1 consisting of 4 subjects treated with placebo and 4 subjects treated with 1 mg/day rapamycin for 4 months, to explore its efficacy and safety; and a phase 2 consisting of 10 subjects treated with placebo and 7 patients treated with 1 mg/day rapamycin for 8 weeks. During phase 1, only one participant treated with rapamycin experienced nocturnal diarrhea after 11 weeks of treatment, which resolved after discontinuation. During phase 2, two subjects, one from the placebo group and the other from the intervention group, reported self-limiting stomatitis lasting a few days. In addition, one subject experienced diarrhea and another suffered from a rash on the face, although the symptoms disappeared in both cases after discontinuation of treatment. Overall, no significant improvements in physical function, as assessed by handgrip strength and walking tests, were observed following treatment with rapamycin, suggesting the need for studies with larger cohorts to verify its effectiveness on musculoskeletal health [109]. Importantly, some studies have thoroughly characterized the pharmacokinetics of rapamycin in the context of kidney transplants, tuberous sclerosis, solid tumors, and lymphomas, but this information may not be applicable to its use in promoting healthy aging. Some evidence has shown that a low dose of rapamycin of 5 mg promotes improvements in the immune system of a cohort of elderly people [110,111]. In this regard, it has been reported that off-label use of rapamycin is fairly widespread in the United States, demonstrating its safety for prolonged treatment, although targeted studies are needed to clarify its pharmacokinetics and bioavailability in the context of healthy aging [112].

Although preclinical evidence has demonstrated the efficacy of quercetin in counteracting the senescence characteristic of age-related musculoskeletal disorders, the results of currently available clinical studies are somewhat limited and contradictory. Farr et al. published the results of a double-blind RCT evaluating the effects of intermittent administration of dasatinib and quercetin in 60 postmenopausal women [113]. Specifically, participants took 100 mg/day dasatinib for two consecutive days and 1000 mg/day quercetin for three consecutive days, with an intermittent schedule repeated every 28 days for 20 weeks. At week 20, serum CTX-1 did not differ between groups, while procollagen type 1 N-terminal propeptide (P1NP) was significantly increased at 2 and 4 weeks, although it did not differ from the control at the end of treatment. In addition, some adverse events were reported by the intervention group, with headache and gastrointestinal events being the most frequent problems. In conclusion, the authors suggested that the dasatinib and quercetin formulation is more effective in stimulating bone formation, like the action of some anabolic drugs such as romosozumab, rather than in inhibiting resorption, highlighting the importance of investigating the effects of further combinations of senotherapeutics in different modes of administration [113]. On the other hand, Bailly et al. conducted a double-blind RCT on 33 postmenopausal women investigating the effects of daily supplementation with 500 mg of quercetin for 90 days on serum levels of osteocalcin, P1NP, CTX-1, and inflammatory markers [114]. Although there were no significant baseline differences between the intervention group and the placebo group, a significant increase in serum levels of these markers was found at the end of treatment. In addition, a significant reduction was found for IL-6 and TNF-α, highlighting the anti-inflammatory and anti-senescence potential of quercetin. However, caution is required when interpreting the results, as the increase in the bone resorption marker does not clarify whether quercetin can protect against bone loss [114]. Unfortunately, evidence regarding the muscular effects of quercetin supplementation in elderly populations is rather scarce, limiting our understanding of the benefits of this flavonoid in the context of sarcopenia. Nevertheless, Nishikawa et al. conducted a double-blind RCT on 26 elderly individuals to study the effects of daily intake of 200 mg of quercetin glucoside on chronic adaptations of muscle strength and motor unit behavior following 6 weeks of resistance training [115]. Maximal voluntary force (MVF) of knee extensor muscle increased in both groups, although a greater increase was seen in the treated group. Similarly, motor unit discharge rates with recruitment rates between 20 and 40% of MVF and between 40 and 60% of MVF were higher at the end of 6 weeks in the intervention group compared to the placebo group, suggesting that quercetin intake, in combination with resistance training, may promote greater muscle strength gains during aging [115]. However, quercetin is characterized by poor solubility and bioavailability, requiring encapsulation or combination with other polymers. Particularly, encapsulation of quercetin in 20–50 nm nanoparticles appears to increase antioxidant activity compared to free quercetin. In this regard, Georgiou and colleagues summarized the different combinations of quercetin and additives used for the treatment of specific pathological conditions, highlighting the effectiveness of the quercetin plus polyphosphate formulation for the treatment of osteoporosis, as well as the combination of quercetin plus oleuropein for the prevention and treatment of joint disorders [116].

Importantly, Varma et al. conducted a double-blind RCT on 30 subjects ≥ 65 years, administering 500 mg/day bioavailable curcumin for 3 months, observing an increase in handgrip strength and weight lift strength compared to placebo, without finding any significant adverse effects [117]. More recently, Kheiridoost and colleagues conducted a triple-blind RCT involving 120 postmenopausal women aged between 50 and 65 with primary osteoporosis or osteopenia to assess the impact and safety of nanomicellar curcumin, Nigella sativa oil, and their combination on bone turnover biomarkers [118]. Specifically, women were treated with 80 mg nanomicellar curcumin (CUR group), 1000 mg Nigella sativa oil (NS group), or both (CUR-NS group), in addition to therapy with 70 mg alendronate and supplements of 500 mg calcium and 400 IU vitamin D. Serum ALP levels were significantly lower in the CUR-NS group than in the placebo group, highlighting the effectiveness of the combination of curcumin and Nigella sativa oil in counteracting bone resorption. Furthermore, analysis of renal metabolites and liver enzymes did not indicate any toxicity of the doses administered. Noteworthy, since curcumin is characterized by relatively low water solubility and its oral bioavailability is inadequate, limiting its use in clinical studies, encapsulation in nanomicelles could overcome these obstacles by optimizing its beneficial effects [118]. In fact, the core–shell structure of the nanomicelles prevents the penetration and presence of water in their inner core, creating a suitable environment for the encapsulated drug, promoting its stability and reducing its degradation compared to the free drug [119].

Vallibhakara et al. conducted a double-blind RCT providing interesting results on the effects of supplementing 400 IU/day of mixed tocopherol for 12 weeks on bone turnover in 52 postmenopausal women [120]. At the end of the treatment period, a significant increase in serum CTX-1 levels was detected in the placebo group compared to baseline, while levels of this marker were essentially unchanged in the treated group. However, no significant changes in P1NP levels were found between the two groups, suggesting the ineffectiveness of vitamin E supplementation in the context of bone formation. Nevertheless, monitoring of adverse events supports the safety of vitamin E supplementation in postmenopausal women, as only one participant reported vaginal bleeding with spontaneous resolution [120]. Interestingly, the benefits of vitamin E supplementation may also extend to skeletal muscle, as evidenced in the double-blind, placebo-controlled RCT by Bo and colleagues on 60 sarcopenic elderly subjects aged between 60 and 85 years [121]. Specifically, participants were randomized into an intervention group, which received whey protein supplementation, 702 IU/day vitamin D, and 109 mg/day vitamin E for 6 months, and a placebo group, which received an isocaloric control product for the same period. Although no significant differences in appendicular muscle mass and walking speed were observed at the end of the supplementation period, the group undergoing nutritional intervention showed a significant improvement in handgrip strength, relative skeletal mass index (RSMI) measured by bioimpedance, and quality of life, along with a reduction in serum interleukin-2 (IL-2). About adverse events, three participants in the treatment group reported difficulty in defecation, while one subject in the placebo group and one subject in the intervention group reported pain during urination [121]. Undoubtedly, further studies are needed to investigate the musculoskeletal benefits of vitamin E, examining possible methods of administration to increase its bioavailability. In fact, the bioavailability of vitamin E appears to be extremely variable, as it is influenced by various dietary factors, such as food matrix, fats, and fat-soluble micronutrients [122].

Several RCTs have been conducted to evaluate the efficacy of genistein in maintaining bone health [123,124,125]. Of particular interest is the post hoc analysis by Arcoraci et al. on data from a previous clinical trial involving 389 postmenopausal women [126,127]. This analysis was conducted on a cohort of 121 women with osteoporosis, 59 of whom belonged to the placebo group and 62 of whom were treated with 54 mg/day genistein. Importantly, a significant increase in BMD of the femoral neck was already observed after one year of treatment, while a significant reduction in this parameter was observed in the placebo group. Similarly, an increase in BMD in the lumbar spine and total hip was found in women treated with genistein, along with an increase in ALP levels, highlighting the effectiveness of genistein in counteracting the progression of osteoporosis. Considering the entire cohort of patients enrolled in the RCT, the most frequent adverse events were gastrointestinal in nature and occurred predominantly in the genistein-treated group [126,127]. It is important to note that genistein has been described as one of the most active natural flavonoids, capable of exerting various biological effects such as chemoprevention, antioxidant action, and antitumor activity. Although a fair amount of RCTs have been conducted on genistein administration, there are currently no clinical trials available to evaluate its benefits in the context of sarcopenia and physical function in the elderly, suggesting the importance of designing clinical studies aimed at understanding its full potential. However, the critical issue to consider in the design of clinical trials of this type is the low oral bioavailability of genistein, which could limit its effects on the elderly population [128]. In this context, the RCT by Anupongsanugool et al. compared the pharmacokinetics and bioavailability of plasma isoflavones daidzein and genistein after a single dose of soy beverage with that provided by soy extract capsules in a cohort of postmenopausal Thai women. The authors found no significant differences in the pharmacokinetics and bioavailability of genistein from both preparations, highlighting the need to develop strategies to optimize its effects [129].

Unfortunately, there is no clinical evidence available to investigate the effects of fisetin on musculoskeletal conditions in the elderly. Considering the promising preclinical results, the design and development of RCTs based on fisetin supplementation should be a crucial research objective in this field. However, fisetin is characterized by poor bioavailability, a limitation that must be overcome for optimal use. In this regard, Krishnakumar et al. conducted an RCT to study the pharmacokinetics and bioavailability of a particular formulation of fisetin micelles encapsulated in a fenugreek galactomannan hydrogel scaffold, finding a plasma concentration in subjects who had taken this compound that was 26.9 times higher than in subjects treated with unformulated fisetin [130]. This information is extremely relevant for anyone wishing to undertake clinical trials based on the use of fisetin, as several factors, such as low gastric pH conditions and extensive intestinal biotransformation, affect its bioavailability. Encapsulation appears to protect fisetin from degradative factors, increasing release times and intestinal absorption [131].

Important evidence of the beneficial effects of epicatechin on muscle health was provided by Mafi et al., who randomized 62 elderly sarcopenic men aged between 65 and 75 into four groups: one group underwent resistance training (RT), one group was treated with 1 mg/kg/day epicatechin (EP), one group underwent resistance training and epicatechin supplementation (RT + EP), and one group received a placebo [132]. Specifically, each training session consisted of a 10 min warm-up, followed by 45 min of leg press, leg extension, leg curl, chest press, shoulder press, rowing, biceps curl, and sit-ups divided into 3 sets of 8–12 repetitions, with a 90 s rest period between each set. With regard to muscle strength and physical capacity, the authors found a significant reduction in the time required to complete the timed up and go (TUG) test in the RT, EP and RT + EP groups, as well as a significant increase in maximum chest press and leg press strength and a significant reduction in plasma myostatin levels in the RT and RT + EP groups. On the other hand, plasma follistatin levels increased significantly in the RT, EP, and RT + EP groups, in association with an increase in the ratio of follistatin to myostatin levels, suggesting the effectiveness of the combination of resistance exercise and epicatechin in counteracting the myostatin pathway that predisposes to sarcopenia [132]. Noteworthy, although epicatechin has been reported to have good bioavailability, it is important to remember that this property can be influenced by other polyphenols and nutritional components, highlighting the importance of a varied and adequate diet in combination with regular physical exercise [133].

Unfortunately, clinical evidence on the effects of synthetic senotherapeutics in the context of musculoskeletal health is somewhat limited, suggesting the need for clinical trials to evaluate their actual efficacy and tolerability in the elderly population. Nevertheless, relevant information has been provided by Farr and colleagues, who demonstrated the efficacy of the combination of dasatinib and quercetin in promoting bone formation in postmenopausal women [113]. In addition, several clinical studies have been conducted to evaluate the effects of metformin on musculoskeletal metabolism. Among these, the RCT by Qaisar and colleagues aimed to evaluate the physical capacity of 132 men randomized into a placebo group and a group treated with 1700 mg/day metformin for 16 weeks [134]. At the end of treatment, the authors assessed handgrip strength and walking speed, finding a significant increase in both parameters in the intervention group, while no significant change was observed in the placebo group. On the other hand, the skeletal muscle mass index was not significantly different between the two groups at the end of treatment, nor were any changes observed in sarcopenia phenotypes between participants in the placebo group and those in the intervention group. Furthermore, the assessment of physical function using the short physical performance battery (SPPB) provided encouraging results, as significantly higher scores were found in the treated group. Interestingly, the authors measured plasma biomarkers associated with neuromuscular junction degeneration, including c-terminal agrin-fragment-22 (CAF22) and neurofilament light chain (NfL), finding a significant reduction in the treated group. Among the adverse events, flatulence was reported in 7 subjects, soft stools/diarrhea in 9 subjects, and a metallic taste in 5 subjects in the treated group. Overall, these results highlight the efficacy of metformin in improving physical function in older adults, likely due to stabilization of the neuromuscular junction, and suggest the need for further investigation of the clinical relevance of metformin in promoting muscle health and healthy aging [134]. In fact, most clinical evidence investigating the effects of metformin on musculoskeletal health focuses on populations of individuals with comorbidities, especially diabetes and cancer, limiting its evaluation in the context of healthy aging [135,136]. Furthermore, one critical issue to be addressed concerns its very low bioavailability, in addition to a short half-life and a narrow absorption window. Therefore, it is necessary to develop delivery systems that improve the absorption of metformin, as current ones are limited by long latency times and the use of non-biocompatible excipients [137].

**Table 4:** A schematic representation of clinical evidence on the efficacy and safety of the main senotherapeutics investigated and/or combined with physical exercise.

Compound	Study Type	Study Population	Evidence	Adverse Events	Reference
**Resveratrol**	Double-blind RCT	*n* = 30 older adults aged 65–80 years underwent to resistance training for 12 weeks: - Placebo group (*n* = 6 males, *n* = 9 females) - Treated group (*n* = 6 males, *n* = 9 females): 500 mg/day resveratrol for 12 weeks	In the treated group: - Improved mitochondrial density and muscle fatigue resistance - Increased fiber area and number of myonuclei in the vastus lateralis muscle	No adverse events reported during the study	[102]
	Three-arm, two-site pilot RCT	*n* = 60 older adults ≥ 65 years with physical limitations underwent to two sessions a week for 12 weeks of center-based walking and whole-body resistance training: - EX0 group (*n* = 6 males, *n* = 14 females): placebo for 12 weeks - EX500 group (*n* = 7 males, *n* = 13 females): 500 mg/day resveratrol for 12 weeks - EX1000 group (*n* = 2 males, *n* = 18 females): 1000 mg/day resveratrol for 12 weeks	In the EX1000 group: - Clinically significant improvement in the walking endurance - Increased citrate synthase	27 adverse events (EX0, *n* = 8; EX500, *n* = 12; EX1000, *n* = 7) related to gastrointestinal or musculoskeletal problems	[103]
	Double-blind crossover RCT	*n* = 128 postmenopausal women ≥ 65 years - Placebo group (*n* = 65) - Treated group (*n* = 63): 75 mg/day resveratrol for 12 months	In the treated group: - Increased BMD in the lumbar spine, femoral neck and total hip - Reduced plasma CTX-1	Treated group: 4 subjects developed itching, menses, prolapsed bladder, prescheduled eye operation	[104]
	Double-blind RCT	*n* = 92 knee osteoarthritic patients, average age 58 years - Placebo group (*n* = 10 males, *n* = 32 females): 15 mg meloxicam + placebo, once daily for 90 days - Treated group (*n* = 13 males, *n* = 37 females): 15 mg meloxicam + 500 mg resveratrol, once daily for 90 days	In the treated group: - Improvement in pain and physical function	No serious adverse events reported during the study	[106]
**Rapamycin**	Double-blind RCT	*n* = 114 healthy individuals aged 50–85 years: - Placebo group (*n* = 24 males, *n* = 15 females) - 5 mg/week of rapamycin (*n* = 23 males, *n* = 17 females) for 48 weeks - 10 mg/week of rapamycin (*n* = 27 males, *n* = 8 females) for 48 weeks	- Improvement in lean body mass and self-reported pain in the 10 mg/week rapamycin group - Improvement in self-reported emotional well-being and overall health in the 5 mg/week rapamycin group	Similar adverse events between groups, with a higher frequency of gastrointestinal symptoms in the treated groups	[108]
	Double-blind RCT	25 healthy older adults aged 70–95 years Phase 1: - Placebo group (*n* = 4 males) - Treated group (*n* = 4 males): 1 mg/die rapamycin for 4 months Phase 2: - Placebo group (*n* = 5 males, *n* = 5 females) - Treated group (*n* = 5 males, *n* = 2 females): 1 mg/die rapamycin for 8 weeks	No significant differences in cognitive function, physical performance, or self-perceived health status between the experimental groups	- Phase 1: one subject treated with rapamycin developed nocturnal diarrhea after 11 weeks of treatment Phase 2 - Placebo group: one subject with self-limiting stomatitis - Treated group: three subjects developed, respectively, self-limiting stomatitis, diarrhea, and facial rash	[109]
**Dasatinib** **Quercetin**	Double-blind RCT	*n* = 60 postmenopausal women aged 60–90 years: - Control group (*n* = 30) - Treated group (*n* = 30): 100 mg/day dasatinib for two consecutive days + 1000 mg/day quercetin for three consecutive days orally with an intermittent schedule repeated every 28 days for 20 weeks	- Increased serum P1NP at 2 and 4 weeks in the treated group - No significant differences in serum CTX-1 between the two groups	Headaches and gastrointestinal events were more frequent in the treated group	[113]
**Quercetin**	Double-blind RCT	*n* = 33 postmenopausal women aged 45–75 years: - Placebo group (*n* = 18) - Treated group (*n* = 15): 500 mg/day quercetin for 90 days	In the treated group: - Increased serum levels of osteocalcin, P1NP and CTX-1 - Reduced serum levels of IL-6 and TNF-α	No adverse events reported during the study	[114]
	Double-blind RCT	*n* = 26 older adults aged 65–82 years undergoing to isometric knee extension resistance training on both legs three times a week for 6 weeks: - Placebo group (*n* = 5 males, *n* = 8 females) - Treated group (*n* = 6 males, *n* = 7 females): 200 mg/day quercetin glycosides for 6 weeks	In the treated group: - Increased MVF of the knee extensor muscle - Increased motor unit firing rates with recruitment thresholds between 20 and 40% of MVF and between 40 and 60% of MVF	No adverse events reported during the study	[115]
**Curcumin**	Double-blind RCT	*n* = 30 healthy older adults ≥ 65 years (*n* = 13 males, *n* = 17 females) - Placebo group (*n* = 15) - Treated group (*n* = 15): 500 mg/day curcumin for 3 months	In the treated group: - Increased handgrip strength and weight lift strength	No serious adverse events reported during the study	[117]
	Triple-blind RCT	*n* = 120 postmenopausal women aged 50–65 years treated with 70 mg alendronate: - Placebo group (*n* = 30): placebo of nanomicelle curcumin + placebo of Nigella sativa oil once a day for 6 months - CUR group (*n* = 30): 80 mg nanomicelle curcumin + placebo of Nigella sativa oil once a day for 6 months - NS group (*n* = 30): placebo of nanomicelle curcumin + 1000 mg Nigella sativa oil once a day for 6 months - CUR-NS group (*n* = 30): 80 mg nanomicelle curcumin + 1000 mg Nigella sativa oil once a day for 6 months	- Reduced serum ALP in the CUR-NS group	No adverse events reported during the study	[118]
**Vitamin E**	Double-blind RCT	*n* = 52 postmenopausal osteopenic women aged over 45-year-old: - Placebo group (*n* = 26) - Treated group (*n* = 26): 400 IU/day mixed-tocopherol for 12 weeks	- Significant increase of 35.3% in CTX-1 at 12 weeks of supplementation compared to baseline in the placebo group - No significant change in CTX-1 and P1NP levels in the treated group	Postmenopausal bleeding after 10 weeks of supplementation in one participant, with spontaneous relief	[120]
	Double-blind RCT	*n* = 60 sarcopenic older adult subjects aged 60–85 years: - Placebo group (*n* = 14 males, *n* = 16 females) - Treated group (*n* = 13 males, *n* = 17 females): 57.5% whey protein, 702 IU vitamin D and 109 mg vitamin E for 6 months	In the treated group: - Improved RSMI and handgrip strength - Reduced serum levels of IL-2 - Improved quality of life	- Difficulty defecating in 3 subjects in the treated group - Pain during urination in one subject in the placebo group and one subject in the treated group	[121]
**Genistein**	Post hoc analysis using data from a multicenter RCT	*n* = 121 postmenopausal osteoporotic women, average age 54 years: - Placebo group (*n* = 59) - Treated group (*n* = 62): 54 mg/day genistein for 24 months	In the treated group: - Increased BMD in the femoral neck, lumbar spine, and total hip at 1 year and 2 years - Increased ALP level	Gastrointestinal problems were the most common adverse event after treatment	[126,127]
**Epicatechin**	Double-blind RCT	*n* = 62 sarcopenic elderly males aged 65–75 years: - Placebo group (*n* = 15) - RT group (*n* = 14): 3 sets of 8–12 repetitions consisting of 45 min of leg press, leg extension, leg curl, chest press, shoulder press, rowing, biceps curl, and sit-ups, with a 90 s rest between sets, for 8 weeks - EP group (*n* = 17): 1 mg/kg/day epicatechin for 8 weeks - RT + EP group (*n* = 15)	In the RT, EP, and RT + EP groups: - Reduced time to complete the TUG test - Increased plasma follistatin levels - Increased follistatin/myostatin ratio, with higher values in the RT + EP group In the RT and RT + EP groups: - Increased maximum strength of chest press and leg press - Reduced plasma myostatin levels	No adverse events reported during the study	[132]
**Metformin**	RCT	*n* = 132 older adult males ≥ 70 years - Placebo group (*n* = 70) - Treated group (*n* = 62): 1700 mg metformin twice a day for 16 weeks	In the treated group: - Increased handgrip strength, gait speed, and physical performance - Reduced plasma levels of CAF22 and NfL	Treated group: - Metallic taste (*n* = 5) - Soft stools/diarrhea (*n* = 9) - Flatulence (*n* = 7)	[134]

RCT: randomized controlled trial; BMD: bone mineral density; CTX-1: C-terminal telopeptide of type 1 collagen; P1NP: procollagen type 1 N-terminal propeptide; IL-6: interleukin-6; TNF-α: tumor necrosis factor-alfa; MVF: maximal voluntary force; ALP: alkaline phosphatase; RSMI: relative skeletal mass index; IL-2: interleukin-2; TUG: timed up and go; CAF22: c-terminal agrin-fragment-22; NfL: neurofilament light chain.

## 7. Discussion

Cell senescence has emerged as a key event in the pathogenesis of numerous age-related musculoskeletal diseases, including osteoporosis, osteoarthritis, and sarcopenia [138,139]. The identification of specific molecular targets associated with this process has opened new perspectives for the development of anti-aging therapeutic strategies in the field of regenerative medicine. However, the high complexity of the signaling pathways involved in the regulation of cellular senescence constitutes a major barrier to the definition of targeted and effective therapeutic interventions [140]. In this context, a deeper understanding of these pathways and their role in the progression of age-related musculoskeletal diseases is essential, promoting research aimed at clarifying the contribution of the multiple actors involved and evaluating the possibilities of modulation using senotherapeutics and physical exercise [26]. Therefore, we have provided an updated overview of natural and synthetic compounds capable of modulating the processes associated with aging in the main musculoskeletal diseases. In addition, particular attention has been paid to the role of physical exercise as a promising non-pharmacological strategy to counteract cellular senescence, highlighting its potential both in prevention and as a complementary therapeutic approach to conventional therapies.

Natural compounds such as resveratrol, rapamycin, quercetin, curcumin, vitamin E, genistein, fisetin, and epicatechin are distinguished by their antioxidant, anti-inflammatory and senotherapeutic properties, acting on key molecular targets such as the AMPK, SIRT1/FoxO, mTOR and NF-κB/β-catenin, as well as regulating the expression of osteogenic factors such as ALP, RUNX2 and Osx [141,142,143,144,145]. Among synthetic compounds, combinations of senolytic agents such as dasatinib and quercetin have been shown to be effective in selectively eliminating senescent cells and reducing the NF-κB-mediated inflammatory response, promoting bone regeneration and slowing the progression of osteoarthritis [64]. However, molecules such as navitoclax and UBX0101 have significant limitations, including side effects on bone formation, cytotoxicity and age-related variability in efficacy, highlighting the need for further research and personalized therapeutic approaches [7,8]. On the other hand, panobinostat has shown potential in activating the FoxO factor [15]; while metformin, considered a promising calorie restriction mimetic, activates the AMPK/SIRT1 pathway, promoting autophagy and reducing oxidative stress, with protective effects on the musculoskeletal system [68,146].

Importantly, available scientific evidence indicates that physical exercise can physiologically modulate numerous molecular targets typically associated with the action of senotherapeutics, helping to improve the balance between proliferation and elimination of senescent cells [147,148,149]. Undoubtedly, this process is potentially relevant in mitigating the progression of diseases associated with cellular aging. Among the main mechanisms involved, the activation of the AMPK signaling pathway induced by physical exercise and the improvement of the NAD^+^/NADH ratio play a key role in maintaining energy homeostasis and regulating cell survival and reprogramming [150,151]. In addition, exercise exerts a regulatory effect on key proteins in the senescence process, such as p16, p21 and p53, influencing cell cycle arrest and apoptosis pathways [24]. Another pathway involved is the AMPK/FoxO3a axis, whose activation promotes autophagy and mitochondrial function, favoring cell survival. In this context, the increase in Bcl-2 expression and the concomitant reduction in Bax suggest a cytoprotective role for physical exercise, through the inhibition of excessive apoptosis [98,99].

Noteworthy, exercise is known to activate SIRT1, an NAD^+^-dependent deacetylase that interacts with transcriptional factors such as PGC-1α, FoxO, and NF-κB, regulating the response to oxidative stress, energy metabolism, and cellular senescence [152]. Among these, PGC-1α acts as a coactivator of ERRα, enhancing its regulatory activity on the expression of genes involved in mitochondrial biogenesis [153,154]. The increase in SIRT1 expression induced by exercise is also associated with the restoration of NAD^+^ and NAMPT levels, suggesting greater efficiency in the cellular response to oxidative stress [155]. In addition, regular exercise appears to promote the expression of other sirtuins, including SIRT3, which is responsible for regulating mitochondrial metabolism, and SIRT6, which is involved in DNA repair and genomic stability, with a positive impact on cellular longevity [84].

Interestingly, the PI3K/Akt signaling pathway, which is essential for muscle regeneration and modulated by growth factors such as IGF-1, BDNF and EGF, is positively influenced by physical exercise [156,157]. In addition, exercise promotes the expression of FNDC5, a precursor of irisin, a myokine involved in communication between bones and muscles, and reduces the expression of NOX4, associated with ROS production [85,158]. Finally, a role for physical exercise has been proposed in the activation of NRF2, the main endogenous regulator of antioxidant defenses, which, by interacting with the Drp1 protein, appears to reduce cellular senescence and reverse the sarcopenic phenotype associated with aging [159].

**Figure 3:**
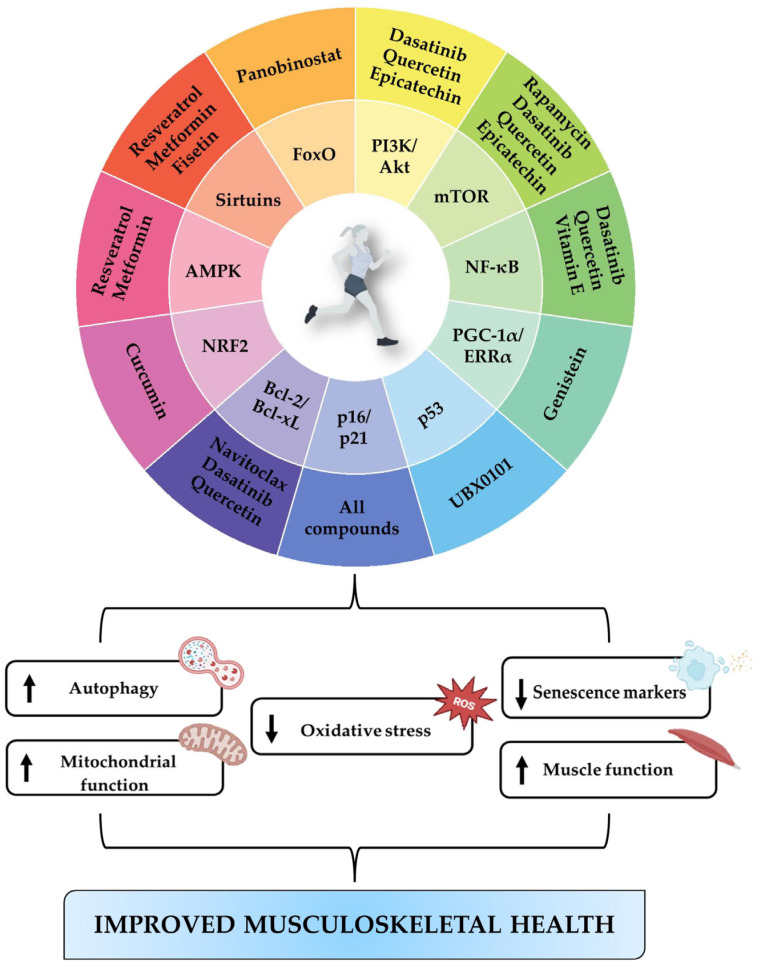
Potential strategies to counteract musculoskeletal aging. Physical exercise is an effective intervention for preserving musculoskeletal health during aging, thanks to its ability to modulate numerous pathways involved in cellular senescence, including phosphoinositide 3-kinase (PI3K)/Akt, mechanistic target of rapamycin (mTOR), nuclear factor kappa-light-chain-enhancer of activated B cells (NF-κB) and peroxisome proliferator-activated receptor gamma coactivator 1-alpha (PGC-1α)/estrogen-related receptor alpha (ERRα). In addition, physical exercise can modulate the expression of cell cycle regulators, including p16, p21, and p53, as well as anti-apoptotic proteins, such as B-cell lymphoma 2 (Bcl-2) and B-cell lymphoma-extra large (Bcl-xL). Finally, physical exercise is known to regulate the expression of factors associated with oxidative stress response and energy metabolism, such as nuclear factor erythroid 2-related factor 2 (NRF2), AMP-activated protein kinase (AMPK), sirtuins, and forkhead box O (FoxO). These molecular pathways are also targets of action for numerous senotherapeutics, including natural compounds such as resveratrol, rapamycin, quercetin, curcumin, vitamin E, genistein, fisetin, and epicatechin, and synthetic compounds, including dasatinib, navitoclax, UBX0101, panobinostat, and metformin (up arrow: increase, down arrow: decrease).

In conclusion, numerous evidence has shown that senotherapeutics and physical exercise represent distinct but potentially synergistic strategies in modulating cellular senescence and managing age-related musculoskeletal disorders (Figure 3). Senotherapeutics, through the selective induction of senescent cell death or the suppression of SASP, show remarkable efficacy in restoring tissue function, although their use is still limited by side effects, the heterogeneity of response, and incomplete evidence of long-term safety. At the same time, physical exercise is a key modulator of numerous molecular pathways involved in cellular homeostasis and energy metabolism, with significant effects on cell survival, autophagy, and mitochondrial biogenesis. Undoubtedly, the integration of these approaches represents a promising direction for promoting healthy musculoskeletal aging. However, careful evaluation is needed to understand the effects of combining exercise and senotherapeutics. Further research will need to evaluate optimal dosing regimens for senotherapeutics to best exploit the long-lasting effects of exercise. In addition, most of the currently available clinical studies on the effects of geroprotectors on musculoskeletal aging are aimed at specific target populations, such as postmenopausal women or frail elderly individuals. Since the sex of the study population may influence the efficacy of senotherapeutics, further studies including male and female cohorts should be conducted to develop optimal strategies for specific populations [149]. Another critical issue to be addressed concerns the low bioavailability of most of the compounds analyzed, which suggests the importance and necessity of developing transport and absorption systems capable of optimizing the effects of senotherapeutics. Notably, Nieman et al. reported that 20 min of vigorous exercise can increase intestinal permeability, accelerating the movement of beneficial phenols from the intestine to the blood. Alternatively, the increase in bioavailability of compounds associated with exercise may depend on the modulation of transporters that control their movement through the intestinal wall, leading to an increase in plasma levels of the compounds ingested [160]. Importantly, although it is universally accepted that regular exercise can be recommended to promote healthy aging, partial adherence to the training program could limit its beneficial effects, thereby negating the benefits of combination therapy. Finally, despite the abundant evidence, it is still unclear which type of exercise is most suitable for preserving musculoskeletal health during aging, highlighting the need for studies comparing the effectiveness of different types of exercise in combination with senotherapeutics. Therefore, strategies based on the combination of physical exercise and senotherapeutics could offer the possibility of promoting healthy aging, but some questions remain open, emphasizing the need for further clinical evidence to clarify the synergistic mechanisms of treatment, efficacy, and long-term safety.

## Data Availability

No new data were created or analyzed in this study. Data sharing is not applicable to this article.

## Abbreviations

The following abbreviations are used in this manuscript:

SASP	Senescence-associated secretory phenotype
mTOR	Mechanistic target of rapamycin
NF-κB	Nuclear factor kappa-light-chain-enhancer of activated B cells
SIRT1	Sirtuin 1
ERRα	Estrogen-related receptor alpha
FoxO1	Forkhead box O1
MSCs	Mesenchymal stem cells
AICAR	5-aminoimidazole-4-carboxamide ribonucleotide
TNF-α	Tumor necrosis factor-alfa
AMPK	AMP-activated protein kinase
RUNX2	Runt-related transcription factor 2
FoxO3	Forkhead box O3
ROS	Reactive oxygen species
ALP	Alkaline phosphatase
OCN	Osteocalcin
OPN	Osteopontin
Col-I	Type I collagen
Osx	Osterix
SOD	Superoxide dismutase
Bcl-2	B-cell lymphoma 2
Bax	Bcl-2-associated X protein
mTORC1	mTOR complex 1
GDF15	Growth differentiation factor 15
STAT3	Signal transduced and activator of transcription 3
BMD	Bone mineral density
CTX-1	C-terminal telopeptide of type 1 collagen
RPMs	Rapamycin-loaded poly(lactic-co-glycolic acid) microparticles
BMSCs	Bone marrow stem cells
VEGF	Vascular endothelial growth factor
ANG-1	Angiopoietin-1
ERK	Extracellular signal-regulated kinase
ITGB1	Integrin subunit beta 1
FAK	Focal adhesion kinase
IGF-1R	Insulin-like growth factor 1 receptor
NRF2	Nuclear factor erythroid 2-related factor 2
IL-4	Interleukin-4
IL-10	Interleukin-10
CD206	Cluster of differentiation 206
IL-1β	Interleukin-1 beta
CCR7	C-C motif chemokine receptor 7
iNOS	Inducible nitric oxide synthase
ATF	α-tocopherol
TRF	Tocotrienol-rich fraction
NAC	N-acetylcysteine
GSH/GSSG	Reduced/oxidized glutathione
SIRT3	Sirtuin 3
PGC-1α	Peroxisome proliferator-activated receptor gamma coactivator 1-alpha
IL-6	Interleukin-6
TGF-β	Transforming growth factor beta
NO	Nitric oxide
PGE2	Prostaglandin E2
COX-2	Cyclooxygenase-2
MMP-3	Matrix metalloproteinase-3
MMP-13	Matrix metalloproteinase-13
ADAMTS-5	A disintegrin and metalloproteinase with thrombospondin motifs 5
IGF-1	Insulin-like growth factor 1
MuRF1	Muscle ring-finger protein-1
SMAD	Suppressor of mothers against decapentaplegic homolog
PI3K	Phosphoinositide 3-kinase
Bcl-xL	B-cell lymphoma-extra large
BMP-2	Morphogenetic protein 2
PRG4	Proteoglycan 4
ACAN	Aggrecan
NOS2	Nitric oxide synthase
OPG	Osteoprotegerin
RANKL	Receptor activator of nuclear factor kappa-B ligand
NAD^+^/NADH	Nicotinamide adenine dinucleotide/reduced NAD^+^
SA-β-gal	Senescence-associated β-galactosidase
MDA	Malondialdehyde
NAMPT	Nicotinamide phosphoribosyltransferase
UCP3	Uncoupling protein 3
HIF-1α	Hypoxia-inducible factor 1-alpha
WBV	Whole body vibration
FNDC5	Fibronectin type III domain-containing protein 5
NOX4	NADPH oxidase 4
CAT	Catalase
SOD2	Superoxide dismutase 2
BAIBA	β-aminoisobutyric acid
BDNF	Brain-derived neurotrophic factor
EGF	Epidermal growth factor
LC3	Microtubule-associated protein 1A/1B-light chain 3
Mfn2	Mitofusin 2
Drp1	Dynamin-related protein 1
PINK1	PTEN-induced kinase 1
RCT	Randomized controlled trial
NSAIDs	Non-steroidal anti-inflammatory drugs
P1NP	Procollagen type 1 N-terminal propeptide
MVF	Maximal voluntary force
RSMI	Relative skeletal mass index
IL-2	Interleukin-2
TUG	Timed up and go
SPPB	Short physical performance battery
CAF22	C-terminal agrin-fragment-22
NfL	Neurofilament light chain
